# Donations of consumables and surgical instruments: how to ensure you really benefit

**Published:** 2011-12

**Authors:** Ismael Cordero

**Affiliations:** Clinical Engineer. Email: ismaelcordero@me.com

**Figure:**
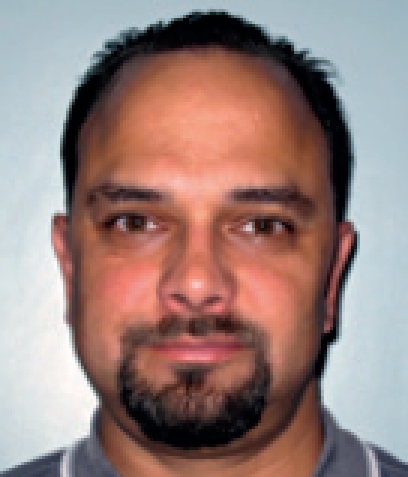
Ismael Cordero

Our “Equipment for eye care” issue (number 73, September 2010) described how donations of equipment can help achieve the goals of VISION 2020 when both donors and recipients follow some general essential guidelines By following the additional suggestions in this issue, you can help to ensure that your unit benefits the most from donated medicines, medical consumables, and surgical instruments.

## General principles

- Ask the donor to provide a list of the medicines, instruments, and consumables they intend to donate so that you can select only those that you really need and which are appropriate to your practice.
- Before accepting any donations of instruments or consumables, make sure that your eye care unit has the appropriate methods, tools, chemical solutions, and equipment in place which are required to clean, sterilise, package, store, and transport these items. Some medicines may have to be refrigerated.
- Do not accept used devices and consumables that are labeled “for single use” since they may not clean and re-sterilise properly. The mechanical integrity and functionality of some single-use devices may suffer when they are cleaned and sterilised.
- Only accept medicines or consumables if you know what the expiry date is and you are sure you will be able to use them before they expire. Ideally, accept only consumables with an expiry date of over one year. This is the standard recommended by the World Health Organization (WHO). In all cases, the date of arrival, the quantities involved, and any expiry dates should be communicated to you well in advance.
- Ask the donor to provide a list of the instruments, consumables, and medicines they intend to donate so that you can select only those that are needed and appropriate to your practice. Refer to the IAPB Standard List for additional guidance (see page 30).
- Only accept donations of items that conform to your country's regulations.

## Medicines

- The presentation, strength, and formulation of donated medicines should be similar to those of the medicines commonly used in your country and unit.
- All donated medicines should be obtained from reliable sources and comply with quality standards in both the donor country and your country.
- You should not accept medicines that have been issued to patients and then returned to a pharmacy or elsewhere, or that were given to health professionals as free samples.
- All medicines should be labeled in a language that is easily understood in your country; the label on each individual container should contain, at minimum, the International Nonproprietary Name (INN) or generic name, the batch number, dosage form, strength, name of manufacturer, quantity in the container, storage conditions, and expiration date.
- Donated pharmaceuticals should be presented in unit sizes and packaging that conform to local regulations.

## Consumables

- For consumables that are intended for use with specific medical devices, such as tubing and cassettes, provide the donor with the specific brand and models of your equipment to ensure that the consumables are a correct match.
- Since the storage and transport conditions of donated consumables may be unknown, it is a good idea for you to inspect the packaging of sterile items to make sure that it is still sealed. It may be difficult for you to know whether the consumable packages have been subject to changes in temperature and humidity outside the storage and transport ranges stated on the package labels. If you have any doubts about an item's sterility, you should discard the item or sterilise it, if it is meant to be sterilised.
- As with medicines, only accept consumables that have a remaining shelf life of at least one year.
- Avoid accepting donations of used implantable devices, such as intraocular lenses (IOLs). These devices require re-sterilisation and special preparations for reuse, which may compromise the device.

**Figure:**
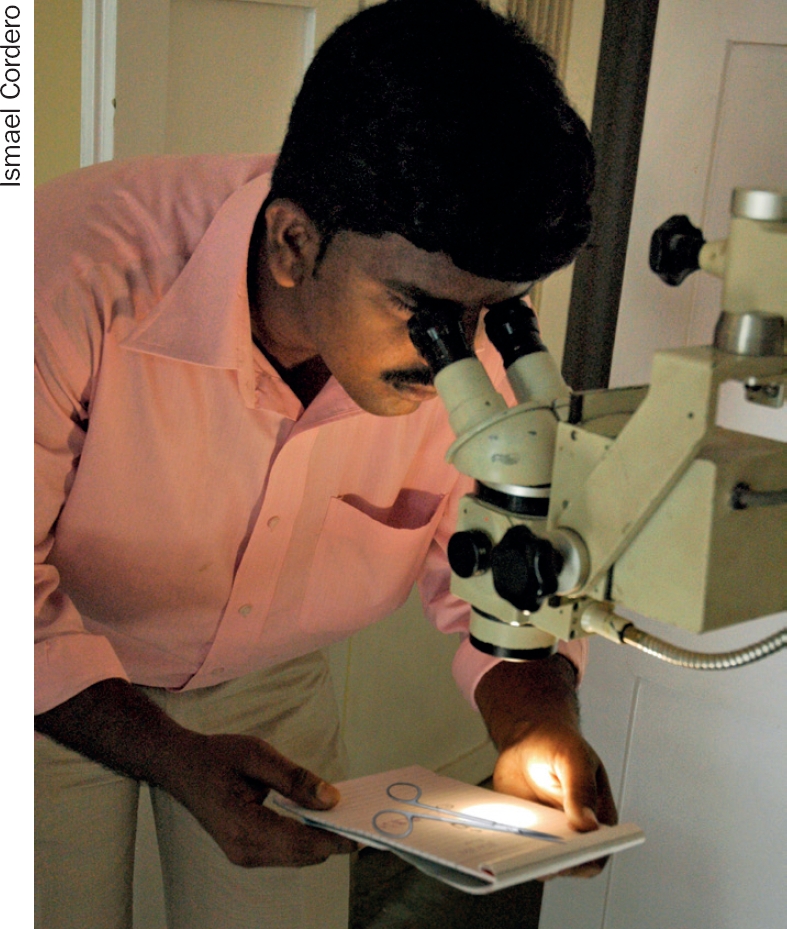
Inspection of instruments should ideally be done with magnification. INDIA

## Surgical instruments

- If you are receiving new types of instruments, make sure that your surgical team is trained in their correct handling and use.
- Single-use instruments should only be accepted if you intend to use them once.
- The quality of surgical instruments varies greatly from manufacturer to manufacturer. Before accepting any instruments, ask for information about the place of origin, manufacturer, standards certifications, and materials used.
- Ask for pictures of the instruments prior to accepting them. This may help you to identify them better since identical instruments can sometimes be known by several different names.
- Ask the donors to pack the instruments for transport so that they do not move around and damage each other.
- Ask the donor to include the manufacturer's instructions for handling, cleaning, and sterilisation.
- Used instruments may be worn to the point where they are misaligned or not sufficiently sharp. If you are accepting a donation of used instruments, make sure that they have been properly maintained prior to donation and that your eye care unit can re-align or sharpen them.
- Donated instruments should always be inspected closely upon receipt. The inspection should ideally be done with magnification and good illumination. If a magnifying glass or a good microscope is not available, a surgical microscope could be used.

